# Quantitative Ultrasound Facilitates the Exploration of Morphological Association of the Long Head Biceps Tendon with Supraspinatus Tendon Full Thickness Tear

**DOI:** 10.1371/journal.pone.0113803

**Published:** 2014-11-20

**Authors:** Ke-Vin Chang, Wen-Shiang Chen, Tyng-Guey Wang, Chen-Yu Hung, Kuo-Liong Chien

**Affiliations:** 1 Department of Physical Medicine and Rehabilitation, National Taiwan University Hospital, BeiHu Branch and National Taiwan University College of Medicine, Taipei, Taiwan; 2 Graduate Institute of Epidemiology and Preventive Medicine, National Taiwan University, Taipei, Taiwan; 3 Department of Physical Medicine and Rehabilitation, National Taiwan University Hospital and National Taiwan University College of Medicine, Taipei, Taiwan; 4 Department of Physical Medicine and Rehabilitation, National Taiwan University Hospital Chu-Tung Branch, Hsinchu, Taiwan; 5 Department of Internal Medicine, National Taiwan University Hospital and National Taiwan University College of Medicine, Taipei, Taiwan; Queen Mary University of London, United Kingdom

## Abstract

**Backgrounds:**

Pathology of the long head biceps tendon (LHBT) is associated with rotator cuff tears but whether the LHBT texture changes following supraspinatus tendon full thickness tear (SSFT) can be detected at the extra-articular segment remains unknown. This cross-sectional study aimed to explore the morphological differences of the LHBT in shoulders with and without deficient rotator cuffs by using quantitative ultrasound.

**Materials and Methods:**

We selected 145 cases with SSFT and 145 age-and- gender-matched controls. The width, thickness, flattening ratio, cross-sectional area, and echogenicity ratio of the LHBT were measured and a general linear model was used to clarify the relationship between rotator cuff pathology and LHBT morphology. The receiver operating characteristic curves of each parameter were constructed for SSFT discrimination and the maximal Youden indexes were used to define the best cut-off points.

**Results:**

We found increased thickness and cross-sectional area but decreased flattening ratio in shoulders with SSFT, and no between-group differences in the width and echogenicity ratio. The LHBT appearance was modified by biceps peritendinous effusion and medial subluxation, but not by the size of SSFT. The flattening ratio was the best discriminator for SSFT with an area under curve of 0.81 (95% confidence interval, 0.76–0.86). The cut-off values to differentiate between the non-tear and tear groups were 2.00 mm of the thickness, 1.73 of the flattening ratio and 10.53 mm^2^ of the cross-sectional area.

**Conclusion:**

Quantitative ultrasound facilitated the detection of the LHBT morphological changes following SSFT and demonstrated its potential utility in discriminating rotator cuff deficiency.

## Introduction

The long head of the biceps tendon (LHBT) is a common origin of anterior shoulder pain and an association between the LHBT pathology and rotator cuff tears has been reported by several arthroscopic case series [1], [2]. Rotator cuff deficiency increases superior translation of the humeral head in relation to the glenoid fossa, causing the LHBT, a humeral head depressor, to be subject to overuse injury [3]. Besides, the LHBT passes through the anterior-superior portion of the rotator cuff tendons, the most prevalent site for tears [4]. Rotator cuff tears of the anterior-superior region, especially in the subscapularis tendon, can tremendously disrupt the stabilizing mechanism of the LHBT, causing medial subluxations or dislocations as well as tendinopathy of the biceps tendon [5].

**Figure 1:**
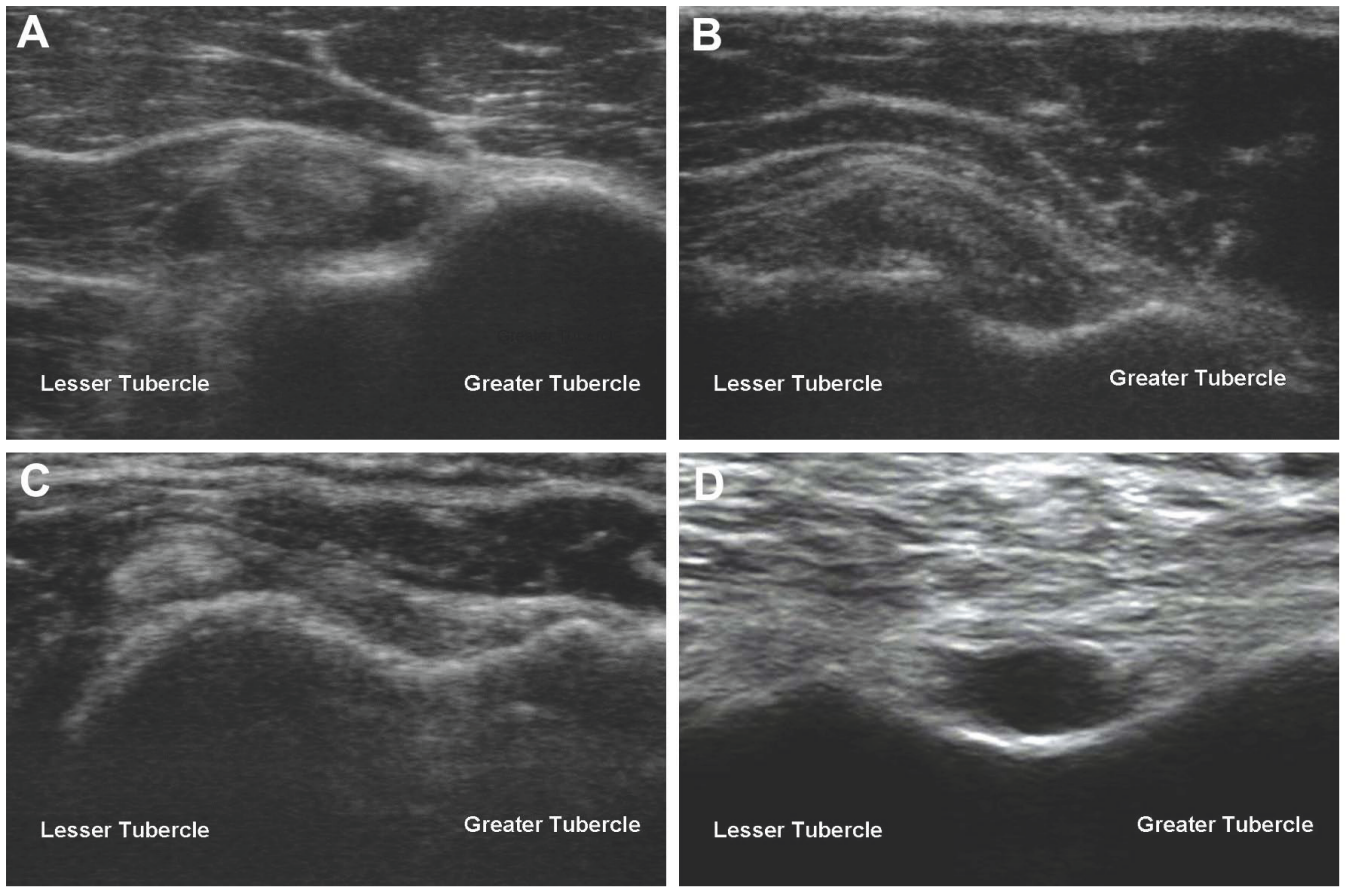
Common pathology of the long head biceps tendons associated with rotator cuff tears: (A) biceps tendinopathy (B) biceps tendon medial subluxation (C) biceps tendon dislocation and (D) biceps tendon tear with an empty bicipital groove.

Among all the biceps pathology concomitant with torn rotator cuffs, tendinopathy is the most common (Fig. 1A) [2]. Clinical symptoms and physical examinations have shown poor sensitivity for detecting biceps tendinopathy and ultrasound has been recommended as the first-line diagnostic image modality [6], [7]. However, the lack of useful quantitative criteria usually makes the sonographic diagnosis of biceps tendinopathy vary between investigators. Although a previous study defined normal values based on the distribution of biceps tendon measurements in a healthy population, the results were not validated by consideration of the coexisting rotator cuff pathology [8]. A recent study indicated that the intra-articular region of the LHBT was more vulnerable to degeneration than its extra-articular segment [9]. Whether the LHBT texture changes in shoulder with SSFT can be detected at the extra-articular segment using ultrasound remains unknown. In addition, since the LHBT at the bicipital groove is the first structure scanned during a standard ultrasound examination of the shoulders, the examiners will be eager to learn of the association of LHBT morphology with rotator cuff lesions, especially supraspinatus tendon full-thickness tears (SSFT). Therefore, the present study aimed to explore the morphological differences of the LHBT in shoulders with and without deficient rotator cuffs by using quantitative ultrasound and to investigate the utility of the LHBT morphology in discriminating SSFT.

## Materials and Methods

### Participants

We reviewed data from patients referred to our musculoskeletal ultrasound laboratory for the examination of shoulder joints between January 2011 and June 2012. The institutional review board of National Taiwan University Hospital approved the retrospective review of these medical records and waived the requirement for informed consent from the participants because shoulder ultrasound investigation was a routine examination and analyzing the image sets had no risk to violate patient rights. Patient characteristics, age, gender, side of examined shoulder, and summarized clinical and physical manifestations were documented in the report with a check list and image set for recording the presence or absence of certain sonographic abnormalities. The structures were investigated by using a high-frequency 10–14 MHz linear transducer (Toshiba America Medical Systems, 2441 Michelle Dr, Tustin, CA 92780), following the order of the biceps tendon, subscapularis tendon, supraspinatus tendon, infraspinatus tendon, acromioclavicular joint, and posterior shoulder recess. All of the ultrasonographic examinations were conducted by a group of board-certified physiatrists with at least 3 years of experience in performing musculoskeletal ultrasound, following a standard protocol regarding patients' postures and transducer placement [10], [11]. In this protocol, the images of LHBT in the short axis view were obtained at the uppermost position of the transverse humeral ligament. A previous study indicated intra-observer reliability of 0.826 and inter-observer reliability of 0.765 by using the guideline for measuring the dimension, cross-sectional area and power Doppler signal amounts of the LHBT [10].

One experienced physician, independent from the examinations, selected suitable cases and processed the images for analysis. Patients aged >20 years and with a complete shoulder ultrasonographic investigation were included. Patients with systemic rheumatologic diseases, malignancy, major trauma, or history of past surgeries of the examined shoulders, were excluded. All the information was verified by reviewing the electronic medical records in our hospital. The SSFT cases were then retrieved from the musculoskeletal ultrasound registry. Shoulders with a torn biceps tendon or a supraspinatus tendon partial-thickness tear were excluded. After the case group was finalized, an age and gender-matched control group with intact supraspinatus tendons was selected at a 1∶1 (case∶control) ratio.

### Diagnostic criteria

The diagnostic criteria for tendinopathy included swelling of the affected tendon and abnormal tendon echotexture with a heterogeneous hypoechoic appearance. Calcification was diagnosed based on the presence of hyper-echogenic plaques with or without an acoustic shadow. Partial and total displacements of the LHBT out of the bicipital groove were recognized as subluxation and dislocation, respectively (Fig. 1B and 1C). Effusion in the bicipital groove was considered abnormal if the thickness exceeded 1 mm in the short axis view. A focal hypoechoic or anechoic defect in the tendon was suggestive of partial-thickness tendon tear, whereas a deficit extending through the entire thickness or complete absence of tendon was referred to as full-thickness tendon tear. A bursal thickness >2 mm was regarded as a positive finding of bursitis. Dome protrusion >3 mm measured from the bony edge and joint space narrowing with adjacent cortical irregularity were indicative of synovitis and osteoarthritis of acromioclavicular joint, respectively [10], [11].

### Morphology analysis (Fig. 2)

**Figure 2:**
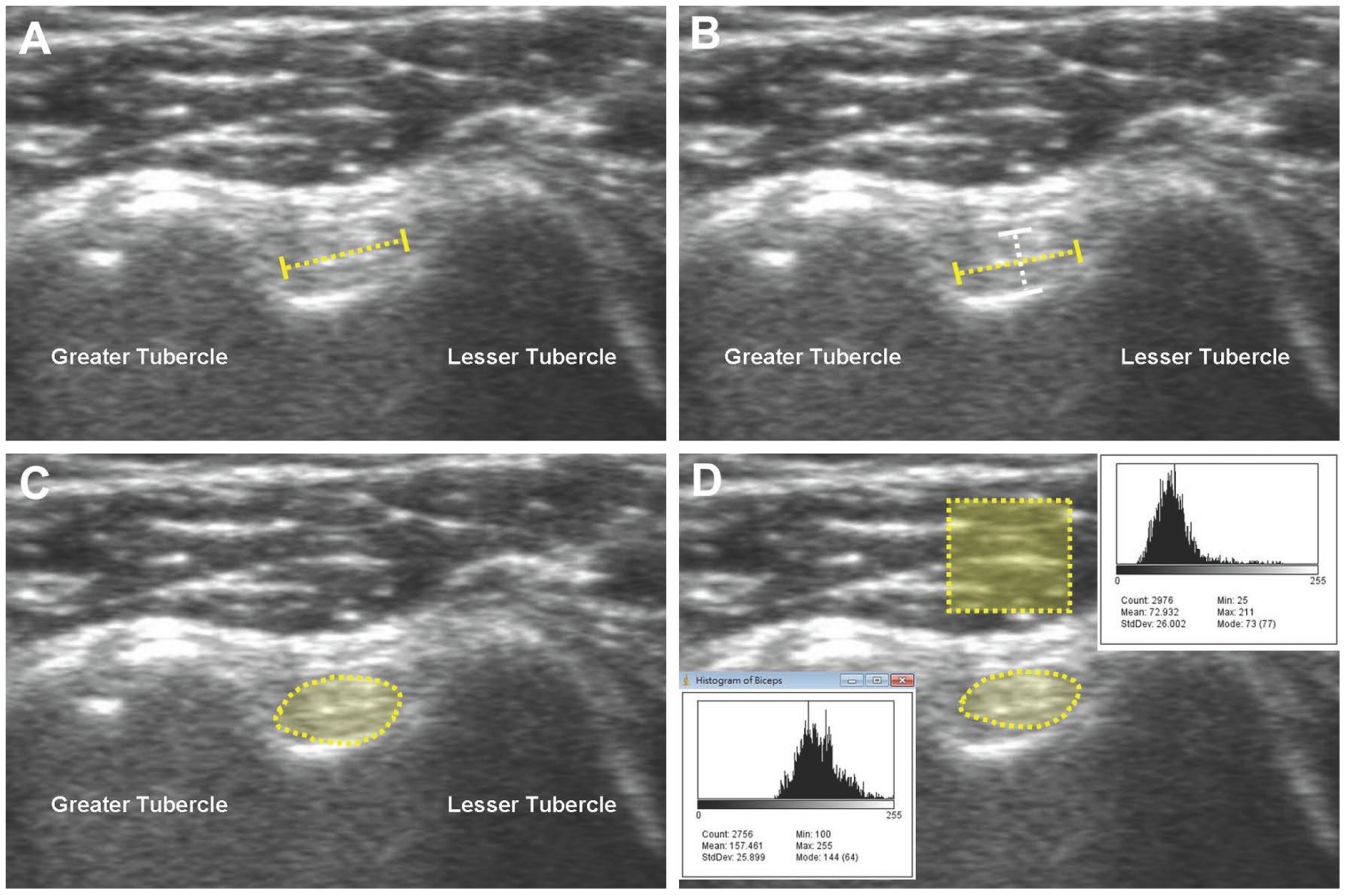
Illustration of how to measure (A) the width (yellow dash line), (B) thickness (white dash line), (C) cross-sectional area, and (D) mean pixel intensity of the long head biceps tendon as well as the mean pixel intensity of the overlying deltoid muscle.

Five parameters, comprising width, thickness, flattening ratio, cross-sectional area, and echogenicity ratio were implemented to depict the morphology of the LHBT and were measured in the short axis view at the bicipital groove level. The width was the maximal diameter of the LHBT (Fig. 2A), whereas the thickness represented the length of a perpendicular axis passing through the middle point of the maximal diameter (Fig. 2B). The flattening ratio indicated the value of the width divided by the thickness. The cross-sectional area was derived from direct tracking of the circumference of the LHBT (Fig. 2C). The average pixel intensity was measured in the entire cross-section of the biceps tendon and a selected square region sized 0.5×0.5 cm in the middle of the deltoid muscle right above the bicipital groove (Fig. 2D). The ratio of the mean pixel intensity of the tendon to that of the reference muscle was defined as the echogenicity ratio [12], [13]. All the measurements were performed using the software Image J (National Institutes of Health, 161 9000 Rockville Pike, Bethesda, MD 20892), with intra-rater reliability of 0.93 and inter-rater reliability of 0.76 expressed by intraclass correlation coefficients according to our previous studies [10], [11].

### Statistical methods

The sample size was estimated from the between-group difference in the thickness of the LHBT. By using a clinically important change of 0.5 mm and a standard deviation of 1 mm, 128 participants were required in each group with a significance level of.05 and a power of 80%. The rationale for assuming 0.5 mm as the between group difference resulted from the measurements of normal LHBTs in a previous study reporting standard reference values for musculoskeletal ultrasonography [8]. In this research, the investigator measured the short axis, long axis and hypoechoic rim of the LHBT. The minimal value among the above mentioned parameters was 0.5 mm, which was present in the measurement of the hypoechoic rim.

Continuous variables and categorical data were compared by using Student's *t*-tests and chi-square tests, respectively. The relative risk was used to estimate the ratio of the probability of biceps tendon tear in the SSFT group compared with that in the group with intact supraspinatus tendons. The multiple linear regression of the general linear model was used to explore the relationship between various kinds of shoulder pathology and the morphological parameters of the LHBT [14]. The independent variables included patients' demographics and shoulders' physical and sonographic findings, whereas the dependent variables consisted of the five parameters of the LHBT morphology. The results were quantified by the coefficients, indicating changes of the morphological parameters in response to a per unit difference in continuous explanatory variables or the shift from a reference status in categorical explanatory variables, holding all other covariates constant. Among the clinical diagnosis of the included patients, we only retrieved the diagnosis of frozen shoulder for model adjustment. Frozen shoulder was diagnosed by ≥50% limitation of shoulder range of motion in any direction for more than 3 months through reviewing the charts documented by the clinicians who referred the cases [15]. Most of them were physiatrists or orthopedic doctors independent from ultrasound examinations.

In addition, the receiver operating characteristic (ROC) curves were constructed to assess the ability of each morphological parameter to discriminate between shoulders with and without SSFT [16]. An area under the curve (AUC) of 0.5 indicates no discriminative value of a diagnostic test. Differences between AUCs were analyzed using the Wald test. The Youden index, a function of sensitivity and specificity, was calculated to find its maximal value for the determination of an optimal cut-off point [16]. Statistical analyses were conducted using the SAS software (version 9.2; SAS Institute Inc., Cary, North Carolina, USA). All p values were two sided and p<.05 indicated statistical significance.

## Results

From a total of 1349 participants referred for an ultrasound examination of the shoulder, SSFTs were identified in 160 shoulders, 15 of which had a concomitant biceps tendon tear. In the overall study population, 17 shoulders were diagnosed with a biceps tendon tear with the relative risk for coexisting with SSFT estimated to be 8.10 (95% CI, 6.42–10.22) compared to that in patients with intact biceps tendons. After excluding shoulders with torn biceps tendons and including 145 age and gender-matched participants with intact rotator cuff tendons, a total of 290 shoulders were included for further morphological analysis of the LHBT.

Table 1 shows the univariate comparisons between the groups with and without a tear (the raw data was provided in File S1). Regarding patients' demographics and physical findings, the group with the tear had more right affected shoulders (p = .03) and a lower prevalence of clinically diagnosed frozen shoulder (p<.01). In terms of the morphological parameters of the LHBT, the shoulders in the group with the tear exhibited an increase in the thickness and cross-sectional area but a decrease in the flattening ratio compared with those in the non-tear group (p of all comparisons <.01). No significant between-group differences were identified in the width and echogenicity ratio (p = .75 and.14, respectively). In regard to the associated sonographic findings, the shoulders with torn supraspinatus tendons demonstrated a greater percentage of biceps peritendinous effusion, and a higher percentage of subscapularis and infraspinatus tendon tears but a lower percentage of supraspinatus tendon calcification (p of all comparisons <.01).

**Table 1:** Participants' demographics and sonographic findings of shoulder joints in the group with supraspinatus tendon full-thickness tears and those without tears.

	The group with intact supraspinatus tendons (n = 145)	The group with supraspinatus tendon full-thickness tear (n = 145)	P value
**Demographics**			
Age, years	67.5±10.7	67.3±10.4	.69
Gender (male), no (%)	54 (37.2%)	54 (37.2%)	1.00
Affected site (right), no (%)	72 (49.6%)	97 (66.8%)	.03*
Frozen shoulder, no (%)	28 (19.3%)	6 (4.13%)	<.01*
**Morphological parameters of the biceps long head tendon**			
Width (mm)	4.60±1.04	4.31±1.07	.75
Thickness (mm)	2.22±0.64	2.83±0.84	<.01*
Flattening ratio (width/thickness)	2.14±0.52	1.57±0.39	<.01*
Cross-sectional area (mm^2^)	10.34±3.95	12.74±6.43	<.01*
Echogenicity ratio	1.91±1.30	1.70±1.24	.14
**Sonographic findings of shoulder**			
Biceps peritendinous effusion, no (%)	59 (40.0%)	104 (71.7%)	<.01*
Biceps medial subluxation, no (%)	2 (1.3%)	7 (4.8%)	.09
Subscapularis tendinopathy, no (%)	26 (17.9%)	39 (26.8%)	.06
Subscapularis tendon calcification, no (%)	11 (7.5%)	20 (13.7%)	.08
Subscapularis tendon tear, no (%)	1 (0.6%)	11 (7.5%)	<.01*
Suprspinatus tendon calcification, no (%)	43 (31.0%)	9 (6.2%)	<.01*
Infraspinatus tendinopathy, no (%)	15 (10.3%)	24 (16.5%)	.12
Infraspinatus tendon calcification, no (%)	7 (4.8%)	9 (6.2%)	.60
Infraspinatus tendon tear, no (%)	0 (0%)	11 (7.5%)	<.01*
Subdeltoid bursitis, no (%)	48 (33.1%)	44 (30.3%)	.61
Posterior recess effusion, no (%)	4 (2.7%)	6 (4.1%)	.52
Acromioclavicular joint synovitis, no (%)	10 (6.8%)	9 (6.2%)	.81
Acromioclavicular joint osteoarthritis, no (%)	14 (9.6%)	19 (13.1%)	.35

The univariate comparisons between both groups were conducted by the Student's t test for continuous variables and the Chi-square test for categorical variables.

Note: continuous variables are expressed as the mean and standard deviation and categorical variables are expressed as the number and percentage in each subgroup.

*indicates a p value less than .05.

Table 2 shows the results derived from the general linear models by employing the thickness, flattening ratio and cross-sectional area as dependent variables, all of which demonstrated significant between-group differences in the univariate analysis. Regarding patients' demographics, being male was positively associated with the flattening ratio and cross-sectional area (p = .05 and.014, respectively), whereas a higher age exhibited a positive association with the cross-sectional area (p<.01). In terms of sonographic findings, SSFT and biceps peritendinous effusion were positively related with the thickness and cross-sectional area but had an inverse association with the flattening ratio. Biceps medial subluxation was positively correlated with the flattening ratio (p = .042). We further dichotomized the study group by using the tear size of 15 mm measured in the transverse view as a cut-off point [4] and found no significant difference between small-to-medium sized and large sized tears on the three morphological parameters of the LHBT.

**Table 2:** Patients' demographics and sonographic findings of shoulder joints associated with the morphological changes of the long head biceps tendons.

	Thickness of the long head biceps tendon		Flattening ratio of the long head biceps tendon		Cross-sectional area of the long head biceps tendon	
	Coefficient	P value	Coefficient	P value	Coefficient	P value
**Demographics**						
Age, years	0.008 (0.004)	.06	0.002 (0.002)	.47	0.089 (0.031)	<.01*
Gender (male vs. female)	0.17 (0.09)	.06	0.12 (0.05)	.04*	1.59 (0.64)	.014*
Affected site (right vs. left)	0.15 (0.09)	.09	−0.07 (0.06)	.17	−0.04 (0.64)	.94
Frozen shoulder (presence vs. absence)	0.15 (0.14)	.26	−0.04 (0.08)	.62	−0.46 (0.99)	.64
**Sonographic findings** (presence vs. absence)						
Supraspinatus tendon full-thickness tear	0.54 (0.10)	<.01*	−0.53 (0.06)	<.01*	1.47 (0.73)	.04*
Supraspinatus tendon calcification	−0.017 (0.123)	.88	−0.017 (0.077)	.82	−1.03 (0.88)	.24
Biceps peritendinous effusion	0.20 (0.09)	.03*	−0.12 (0.06)	.047*	1.69 (0.69)	.014*
Biceps medial subluxation	−0.42 (0.25)	.09	0.32 (0.15)	.042*	0.20 (1.82)	.90
Subscapularis tendinopathy	−0.061 (0.109)	.57	0.009 (0.068)	.89	0.10 (0.78)	.89
Subscapularis tendon calcification	−0.19 (0.14)	.19	0.025 (0.091)	.77	−0.93 (1.04)	.37
Subscapularis tendon tear	−0.27 (0.22)	.21	0.050 (0.142)	.72	−2.47 (1.62)	.18
Infraspinatus tendinopathy	0.17 (0.13)	.20	−0.094 (0.083)	.25	0.87 (0.95)	.35
Infraspinatus tendon calcification	0.09 (0.20)	.66	0.006 (0.130)	.96	2.42 (1.48)	.10
Infraspinatus tendon tear	0.41 (0.23)	.08	0.076 (0.150)	.61	2.80 (1.71)	.10
Subdeltoid bursitis	0.19 (0.09)	.06	−0.063 (0.062)	.30	−0.082 (0.707)	.90
Posterior recess effusion	0.12 (0.24)	.62	−0.28 (0.15)	.06	−0.34 (1.74)	.84
Acromioclavicular joint synovitis	0.17 (0.17)	.34	−0.13 (0.11)	.22	0.66 (1.25)	.59
Acromioclavicular joint osteoarthritis	0.17 (0.14)	.20	−0.015 (0.089)	.86	1.07 (1.01)	.29

The associations were analyzed by a general liner model. Note: the values of coefficients were expressed by point estimates with standard deviations.

*indicates a p value less than .05.

**Figure 3:**
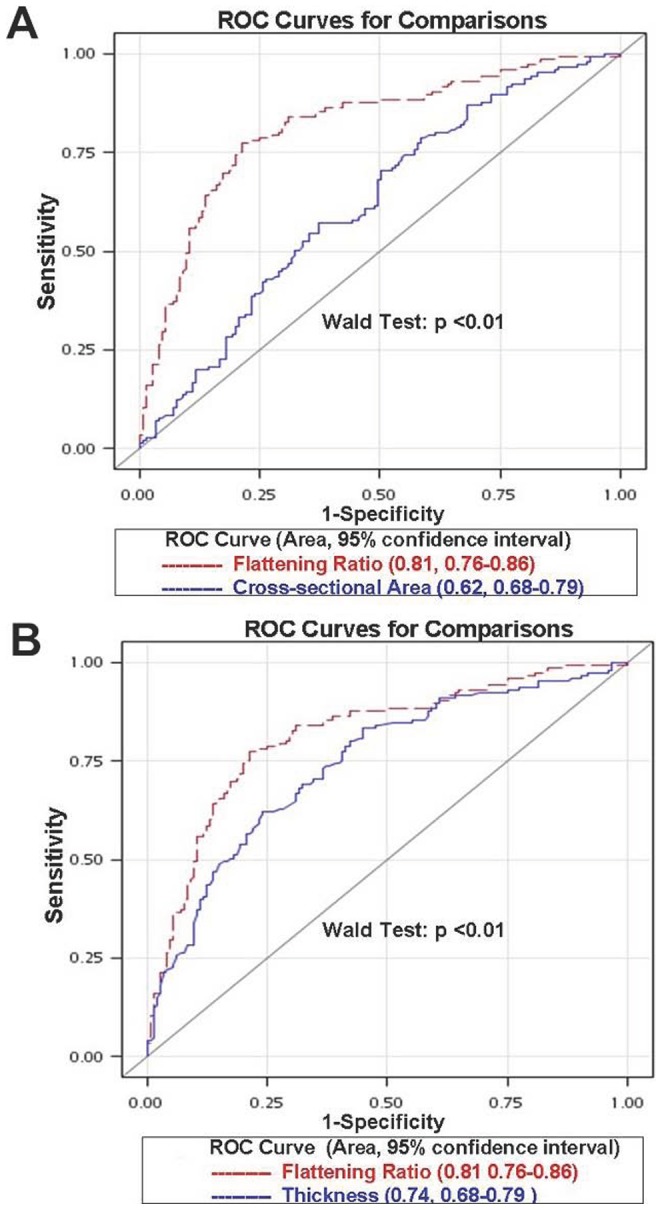
The receiver operating characteristics (ROC) curve of (A) the flattening ratio versus the thickness and (B) the flattening ratio versus the cross-sectional area of the long head biceps tendon in discriminating supraspinatus tendon full thickness tear.

Fig. 3 revealed the results derived from the ROC curve analysis. The AUCs for the thickness, flattening ratio and cross-sectional area to discriminate the presence of SSFT were 0.74 (95% CI, 0.68–0.79), 0.81(95% CI, 0.76–0.86) and 0.62 (95% CI, 0.55–0.68), respectively. The flattening ratio had the best discriminative power compared to the thickness and cross-sectional area (p of both comparisons <.01). Based on the maximal Youden indexes, the cut-off values to differentiate between the non-tear and tear groups were 2.00 mm of the thickness with a sensitivity of 0.62 and a specificity of 0.75, 1.73 of the flattening ratio with a sensitivity of 0.84 and a specificity of 0.68 and 10.53 mm^2^ of the cross-sectional area with a sensitivity of 0.57 and a specificity of 0.75.

## Discussion

The present study quantified the sonographic morphology of the LHBT and explored the differences between shoulders with and without SSFT. The strength of this research included using a clinical registry, the data of which could be generalized to the population in primary care settings. The most novel finding was that the LHBT morphological differences in shoulders with and without SSFT could be identified using ultrasound at the extra-articular segment of the LHBT through a simple process of quantification. We found that the thickness and cross-sectional area of the LHBT significantly increased but the flattening ratio significantly decreased at the bicipital groove level in shoulders with SSFT. The LHBT appearance was modified by biceps peritendinous effusion and medial subluxation, but not by the size of SSFT. Furthermore, the flattening ratio exhibited the best discriminative ability for the presence of SSFT.

We found that the relative risk of SSFT in shoulders with torn biceps tendons reached 8.1 (95% CI, 6.42–10.22) compared with that in patients with intact biceps tendons, implying the LHBT tear (Fig. 1D) a specific sign for SSFT during ultrasound examinations. However, the prevalence of LHBT tears was merely 1.2% (17/1349) in our study population, rendering this finding less useful in discriminating SSFT, the prevalence of which was as high as 11.8% (160/1349).

Evaluation of the LHBT appearance using quantitative ultrasound has been proposed since 2006 and most parameters are related to pixel intensities and distributions in the region of interest [17], [18]. The characteristics of tendinopathy consist of increased skewness and kurtosis, and reduced variance and echogenicity in grayscale images [17], [18]. However, a standard posture of the examinees and a uniform setting of the ultrasound machines are required for the comparisons between pictures. The technique demonstrates a lower inter-rater reliability and is less applicable to clinical conditions. Based on the abovementioned reasons, our study finally decided to use the echogenicity ratio of the LHBT to the reference muscle, a parameter less influenced by machine settings, as the only assessment of tendon echotexture [12], [13]. Besides, we employed modified measurements useful for evaluating the nerves following entrapment [19], such as the flattening ratio and cross-sectional area of the LHBT. As a result, the LHBT morphology could be quantified in three aspects, comprising its size (width, thickness, and cross-sectional area), shape (flattening ratio), and tendon architecture (echogenicity ratio).

The morphological changes of the LHBT, including a significant increase in the thickness and cross-sectional area with a decrease in the flattening ratio, were observed using ultrasound in shoulders with deficient supraspinatus tendons. The findings supported our hypothesis that a tendinopathic change such as swelling and enlargement could be detected in the extra-articular LHBT following SSFT. Normal LHBT is oval-shaped in the transverse view at the bicipital groove and the increase in the short-axis length is more easily identified [20], explaining the reason for being able to detect the discrepancy in the thickness but not the width of the LHBT. Furthermore, the magnitude of the change in shape (being rounder) appeared to be significantly more than the difference in size (being larger), resulting in the flattening ratio being the best parameter to discriminate between shoulders with and without torn supraspinatus tendons. However, although the echogenicity ratio was used to adjust the variance caused by different imaging conditions, the LHBT in shoulders with SSFT was not more hypoechoic. We speculated that the vulnerability of anisotropy in visualizing the LHBT was another potential cause for the estimation of pixel intensities to be a poor discriminator of LHBT tendinopathy.

Our analysis indicated that the morphology of the LHBT in large-sized SSFT did not differ from that in small-to-medium sized tears. Several antecedent studies indicated that the tear size played a less important role than the tear location in terms of shoulder pain and function impairment [21], [22]. Namdari et al found that painful shoulders demonstrated significantly higher rates of anterior supraspinatus tendon tears compared with the asymptomatic rotator-cuff-torn shoulders [4]. This finding might be explained by the proximity of the anterior supraspinatus tendon lesions to the lateral biceps sling, rendering the LHBT vulnerable to instability-associated injury and then eliciting anterior shoulder pain. In our study, we did not categorize the tear group according to the location of SSFT, because not all image sets included views allowing the measurement of the distance from the biceps tendon. Therefore, we could not conclude whether the location affected the LHBT morphology more than the tear size. On the other hand, this result suggested that the investigator should be aware of SSFT of any size if the LHBT morphological parameters deviated from normal ranges.

In the present study, the control group had a higher prevalence of clinically diagnosed frozen shoulder. Although the diagnosis was not confirmed by the golden standard [23], distensibility of joint capsules after intra-articular fluid injection, it was reasonable to speculate that most patients with chronic painful stiff shoulders had primary pathology of joint capsules, not rotator cuff disorders. Therefore, patients with clinically diagnosed frozen shoulder had a higher probability of intact supraspinatus tendons in a painful shoulder population and were likely to be selected as controls.

Several demographic factors and concomitant sonographic abnormalities were shown to modify the LHBT morphology. Age was positively related to the cross-sectional area, which might be related to a tendency of tendon degeneration in the elderly [12]. Male gender was likely to increase the flattening ratio and cross-sectional area; this brought gender difference to the attention of the investigators while interpreting the morphological parameters. Another interesting finding was the bicipital peritendinous effusion, which shifted the thickness, cross-sectional area, and flattening ratio toward the trait of tendinopathy. Because the oval shape of the LHBT in the transverse view at the bicipital groove was partly maintained by the restraint from the transverse humeral ligament, fluid filling the tendon sheath might reduce the compressive pressure, causing the enlargement and conformation changes of the LHBT [24]. However, the results did not contradict our hypothesis that the changes of LHBT morphology were independently associated with SSFT, since the general linear model had adjusted the presence of bicipital peritendinous effusion. Finally, the long axis of the LHBT in the transverse view might be stretched and then elongated in the conditions of subluxation or dislocation (Fig. 1B, 1C), rendering the tendon more flattened. Therefore, the investigators were suggested to treat shoulders with biceps medial subluxation as a separate group if they intended to use the flattening ratio as a discriminative tool for SSFT.

Our result had two potential clinical implications. First, although it seems easy to identify SSFT in well-trained operators, the rule cannot be applied on less-experienced investigators. For anterosuperior rotator cuff tendon tear, the new learners are vulnerable to miss the diagnosis due to inappropriate shoulder positioning [4]. Since the LHBT passes through the anterior-superior portion of the rotator cuff tendons, the most prevalent site for tears, the investigators can take the advantage of the morphological association of LHBT with SSFT to assist the diagnosis. Second, the present study found that the morphological changes of the LHBT were not related to the extent of SSFT. The finding reminds the clinicians of scrutinizing any size of SSFT if the morphological parameters of LHBT exceed the defined value.

### Study limitations

The present research had several limitations. First, we employed a cross-sectional study design, which could merely clarify the association between the LHBT pathological changes and SSFT. We were unable to know how the biceps tendon would change its morphology when the SSFT was left untreated or repaired. A longitudinal follow-up study should be implemented to solve the issue. Second, most participants registered in our musculoskeletal ultrasound registry were symptomatic and our analysis failed to answer whether the association was different in a population without shoulder complaints. We believed that the comparison with the asymptomatic group was of clinical importance, since the grades of LHBT pathology might vary according to their symptom severity. Nevertheless, considering generalization of our results in the hospital-based settings, the use of symptomatic participants without SSFT as controls was still acceptable if the current research aimed at a painful shoulder population referred for ultrasound examinations. Third, our data set did not allow us to explore the association between the location of SSFT and the changes of LHBT morphology, which would be analyzed in our future study using a more standardized measurement of the tear site.

## Conclusions

SSFT was associated with the morphology changes of the extra-articular LHBT examined using ultrasound, comprising an increase in the thickness and cross-sectional area and a reduction in the flattening ratio. Age, gender, biceps peritendinous effusion, and medial subluxation were shown to modify the LHBT appearance, but the difference between shoulders with small-to-medium and large-sized tears were not significant. Among the morphological parameters of the LHBT, the flattening ratio was the best discriminator of SSFT and may be utilized in future large-scale studies for detection of supraspinatus tendon deficiency of different grades.

## Supporting Information

File S1 **Baseline and ultrasound characteristics in participants with and without supraspinatus tendon full thickness tears.** All the information was anonymized.
(XLS)
